# Cobertura vacinal e conhecimentos, atitudes e práticas sobre vacinação contra febre amarela: estudo transversal durante epizootia em São Sebastião, Distrito Federal, 2020

**DOI:** 10.1590/S2237-96222026v35e20250739.pt

**Published:** 2025-11-21

**Authors:** Leonardo José Alves de Freitas, Lairton Souza Borja, Danielle Gonçalves Figueiredo, Fabiano dos Anjos Pereira Martins, Priscilleyne Ouverney Reis, Aline Ruben Cardoso Fernandes Caixeta, Ana Júlia Silva e Alves

**Affiliations:** 1 Ministério da Saúde, Secretaria de Vigilância em Saúde e Ambiente, Programa EpiSUS, Brasília, DF, Brasil; 2 Secretaria de Saúde do Distrito Federal, Núcleo de Vigilância Epidemiológica Leste, Brasília, DF, Brasil; 3 Secretaria de Saúde do Distrito Federal, Subsecretaria de Vigilância à Saúde, Brasília, DF, Brasil; 4 Secretaria de Saúde do Distrito Federal, Diretoria de Vigilância Epidemiológica, Brasília, DF, Brasil; 5 Secretaria de Saúde do Distrito Federal, Diretoria de Vigilância Ambiental, Brasília, DF, Brasil

**Keywords:** Febre Amarela, Vigilância em Saúde Pública, Vacinas, Epidemiologia, Estudos Transversais, Fiebre Amarilla, Vigilancia de la Salud Pública, Vacunas, Epidemiología, Estudios Transversales

## Abstract

**Objetivo:**

Estimar a cobertura vacinal contra febre amarela e descrever conhecimentos, atitudes e práticas (CAP) da população residente em São Sebastião, Distrito Federal, após a confirmação de epizootia em primata não humano na região.

**Métodos:**

Estudo transversal composto por dois inquéritos: um de cobertura vacinal e outro sobre CAP, entre novembro e dezembro de 2020. A amostragem foi probabilística por conglomerados (30x7). Foram considerados vacinados os indivíduos com registro da vacina contra febre amarela em caderneta de vacinação ou no Sistema de Informação do Programa Nacional de Imunizações. Utilizou-se análise complexa e estatística descritiva, com frequências absolutas e relativas, medidas de tendência central e de precisão. Para o estudo de CAP, empregou-se estatística analítica, sendo a razão de prevalência a medida de associação utilizada e intervalos de confiança de 95% (IC95%).

**Resultados:**

Foram realizadas 210 entrevistas. A cobertura vacinal foi de 54,3% (IC95% 47,70; 60,90), com 93,9% dos registros oriundos da caderneta de vacinação. Dos participantes, 63,8% eram do sexo feminino e 60,0% se autodeclararam pardos. Em relação ao componente de CAP, 70,0% apresentaram conhecimentos inadequados, 87,1% atitudes adequadas e 54,3% práticas adequadas. A prevalência de atitudes adequadas entre pessoas com conhecimentos adequados foi 11,10 vezes maior que entre aqueles com conhecimentos inadequados.

**Conclusão:**

O estudo permitiu descrever a cobertura vacinal e CAP relacionados à vacinação contra febre amarela. A cobertura encontrada foi inferior à meta de 95,0% estabelecida pelo Programa Nacional de Imunizações, evidenciando aspectos que podem subsidiar ações de vigilância em saúde e promoção da vacinação.

## Resumen

**Objetivo:**

Estimar la cobertura de vacunación contra la fiebre amarilla y describir los conocimientos, actitudes y prácticas (CAP) de la población residente en São Sebastião, Distrito Federal (Brasil), después de la confirmación de epizootias en primates no humanos en la región.

**Métodos:**

Estudio transversal compuesto por dos cuestionarios: uno sobre la cobertura de vacunación y otro sobre CAP, aplicados entre noviembre y diciembre de 2020. El muestreo fue probabilístico por conglomerados (30x7). Las personas con registro de la vacuna contra la fiebre amarilla en la libreta de vacunación o en el Sistema de Información del Programa Nacional de Inmunización se consideraron vacunadas. Se utilizó análisis complejos y estadísticas descriptivas, con frecuencias absolutas y relativas, medidas de tendencia central y precisión. Para el estudio de CAP, se utilizaron estadísticas analíticas, y como razón de prevalencia se aplicó la medida de asociación y los intervalos de confianza del 95% (IC95%).

**Resultados:**

Se realizaron 210 entrevistas. La cobertura de vacunación fue del 54,3% (IC95% 47,70; 60,90), con un 93,9% de los registros provenientes de la libreta de vacunación. El 63,8% de los participantes son mujeres, y el 60,0% se declaraban pardos. Respecto al componente PAC, el 70,0% presentó conocimientos inadecuados, el 87,1% actitudes adecuadas y el 54,3% prácticas adecuadas. La prevalencia de actitudes adecuadas entre las personas con conocimiento adecuado fue 11,10 veces mayor que entre las personas con conocimiento inadecuado.

**Conclusión:**

Este estudio permitió describir la cobertura de vacunación y CAP relacionados con la vacunación contra la fiebre amarilla. La cobertura encontrada fue inferior al objetivo del 95,0% trazado por el Programa Nacional de Inmunización, lo cual apunta aspectos que pueden apoyar acciones de la vigilancia de la salud y de promoción de la vacunación.

**Aspectos éticos**

Esta pesquisa respeitou os princípios éticos, obtendo os seguintes dados de aprovação:

Comitê de ética em pesquisa: Comissão Nacional de Ética em Pesquisa

Número do parecer: 5.013.116

Data de aprovação: 3/10/2021

Certificado de apresentação de apreciação ética: 49195021.8.0000.0008

Registro do consentimento livre e esclarecido: Na etapa de campo, os participantes assinaram o Termo de Consentimento Livre e Esclarecido (TCLE). Para as análises de dados secundários e anonimizados, foi concedida a dispensa de novo TCLE pela Comissão Nacional de Ética em Pesquisa.

## Introdução

A febre amarela é uma arbovirose aguda, de importância mundial, causada por um vírus do gênero *Flavivirus*. A transmissão ao ser humano ocorre principalmente no ciclo silvestre, por meio da picada de mosquitos dos gêneros *Haemagogus* e *Sabethes*. Os primatas não humanos (PNH) são considerados os principais hospedeiros e amplificadores do vírus no ciclo silvestre da doença ^1^. Embora o Brasil não registre transmissão urbana desde 1942, a ocorrência de epizootias próximas a áreas urbanizadas representa risco de reurbanização da doença e mantém os órgãos de saúde pública em alerta ^2^.

Entre julho de 2019 e junho de 2020, o Brasil notificou 976 casos suspeitos de febre amarela, com 19 casos confirmados e quatro óbitos ^3^. No mesmo período, no Distrito Federal, foram registrados 11 casos suspeitos em humanos, todos descartados. Em novembro de 2020, foi confirmada epizootia em um PNH na região administrativa de São Sebastião ^4^, localizada na porção leste da capital brasileira. O episódio coincidiu com o início do período sazonal da doença, de dezembro a maio, em meio à pandemia de covid-19 ^1^.

A vacinação é a medida mais eficaz para prevenir a febre amarela. Apesar disso, a cobertura vacinal ainda é baixa em várias regiões do Brasil, sobretudo naquelas em transição entre áreas urbanas e rurais ^1^ ^,^ ^5^. Fatores como o desconhecimento sobre a doença, atitudes negativas em relação à vacina e práticas insuficientes de prevenção influenciam diretamente a adesão da população à imunização contra a febre amarela, promovendo a hesitação vacinal ^6^.

Além das barreiras relacionadas à informação, há desafios operacionais nos serviços de saúde, como desabastecimento pontual de vacinas, limitações na busca ativa e dificuldades logísticas para atualização de cadastros e registros vacinais ^7^. Tais fragilidades podem comprometer a efetividade das ações de imunização e favorecer a persistência de bolsões suscetíveis ao vírus da febre amarela mesmo em áreas cobertas pelas campanhas de vacinação. 

Apesar da relevância da febre amarela no Distrito Federal, há escassez de estudos locais que descrevam a cobertura vacinal e os conhecimentos, atitudes e práticas (CAP) da população, principalmente em territórios vulneráveis como São Sebastião. Essa lacuna de informação reforça a importância de inquéritos epidemiológicos para subsidiar e direcionar ações de vigilância em saúde e promoção da imunização, para fins de prevenção e intervenção contra doenças infecciosas.

Nesse contexto, o presente estudo teve como objetivo estimar a cobertura vacinal contra febre amarela e descrever CAP da população residente em São Sebastião, Distrito Federal, após a confirmação de epizootia em PNH na região.

## Métodos

### Delineamento do estudo

Foi realizado estudo transversal de base populacional, composto por dois inquéritos: um de cobertura vacinal contra febre amarela e outro sobre conhecimentos, atitudes e práticas (CAP) em relação à doença e à vacinação. O estudo se originou de uma investigação epidemiológica de campo realizada no período de 25 de novembro a 18 de dezembro de 2020.

### Contexto

Em 9 de novembro de 2020, a Secretaria de Saúde do Distrito Federal (SES-DF) confirmou laboratorialmente a morte de um primata não humano por febre amarela no bairro São José, área urbana da região administrativa de São Sebastião. Em 18 de novembro de 2020, a SES-DF solicitou o apoio da Secretaria de Vigilância em Saúde e Ambiente do Ministério da Saúde (SVSA), por meio do Programa de Treinamento em Epidemiologia Aplicada aos Serviços do Sistema Único de Saúde (EpiSUS), nível Avançado, para investigação epidemiológica de campo.

A investigação foi realizada no território de São Sebastião, região administrativa do Distrito Federal que está localizada na Região de Saúde Leste de Brasília, a 23 km do Plano Piloto. A região apresenta características urbanas e rurais, com população estimada em 115 mil habitantes em 2020, distribuída em 120 setores censitários. O território contava com 14 unidades básicas de saúde (UBS) e cobertura de 78,1% por Equipes de Saúde da Família. As salas fixas de vacinação estavam na UBS 1 e no Sistema Penitenciário, com outras 12 UBS realizando vacinação volante em dias específicos. Os inquéritos foram conduzidos no início do período sazonal da febre amarela, em meio à pandemia de covid-19.

### Participantes

Foi utilizada amostragem seguindo o método probabilístico por conglomerados (30x7), recomendada pela Organização Mundial da Saúde ^8^ ^,^ ^9^. Em cada domicílio sorteado, residentes com mais de nove meses de idade presentes no momento da entrevista foram convidados a participar do inquérito de cobertura vacinal. Para o inquérito CAP, foi selecionado um morador com 18 anos ou mais, definido por sorteio entre os elegíveis presentes no domicílio. Foram excluídos moradores ausentes no momento da visita e recusas. 

Os critérios de inclusão exigiam residência mínima de três meses na região. Foram excluídos imóveis fechados, comerciais, igrejas, unidades prisionais e indivíduos impossibilitados de participar. As definições de vacinado, não vacinado, esquema completo e incompleto, perda e recusa seguiram critérios padronizados ^1^ ^,^ ^10^.

### Variáveis

Foram utilizadas variáveis relacionadas a características sociodemográficas, de vacinação e epidemiológicas. As variáveis sociodemográficas incluíram sexo (feminino, masculino), raça/cor da pele autodeclarada (parda, branca, preta, amarela, indígena, ignorado), escolaridade (superior completo, superior incompleto, médio completo, médio incompleto, fundamental completo, fundamental incompleto, analfabeto, ignorado), idade (em anos), tempo de residência (em anos), ocupação, renda familiar mensal e recebimento de benefícios sociais. 

A variável principal do estudo foi a cobertura vacinal, definida a partir do registro de pelo menos uma dose da vacina contra febre amarela na caderneta de vacinação ou no Sistema de Informação do Programa Nacional de Imunizações (SI-PNI), conforme esquema de vacinação do Ministério da Saúde. A cobertura foi estimada como a proporção de entrevistados com registro comprovado da vacina em relação ao total da amostra. Também foram analisadas variáveis epidemiológicas, incluindo acesso a serviços de saúde, visita a áreas de mata ou cachoeira, motivações e barreiras para vacinação, local de vacinação e fontes de informação.

No inquérito CAP, os conhecimentos foram avaliados a partir de seis perguntas relacionadas à doença e à vacina (o que é a febre amarela, como ocorre a transmissão, formas de proteção, finalidade da vacina, público-alvo e número de doses recomendadas para adultos). Os entrevistados que responderam corretamente a pelo menos quatro dessas questões (66,7%) foram classificados como tendo conhecimentos adequados. As atitudes foram mensuradas por seis perguntas referentes à percepção de risco da doença, prevenção, busca por informações, intenção de vacinar-se, confiança na segurança da vacina e incentivo à vacinação de familiares, sendo consideradas adequadas quando havia pelo menos quatro respostas positivas. A prática foi definida pela situação vacinal, sendo classificada como adequada quando o participante apresentou ao menos uma dose registrada em caderneta de vacinação ou no SI-PNI.

Variáveis derivadas foram utilizadas segundo a classificação do Instituto Brasileiro de Geografia e Estatística (IBGE). A escolaridade foi categorizada em baixa (analfabeto, ensino fundamental incompleto ou completo, ensino médio incompleto) e alta (ensino médio completo, ensino superior incompleto ou completo). Já a renda familiar mensal foi categorizada em baixa (até dois salários mínimos) e média/alta (de três a dez salários mínimos).

### Fontes de dados e mensuração

Durante a investigação de campo, foram conduzidas entrevistas presenciais domiciliares, aplicadas com questionário semiestruturado, previamente testado e padronizado. O instrumento utilizado foi elaborado pela equipe da investigação, com base em instrumentos de CAP previamente aplicados em inquéritos conduzidos no âmbito do EpiSUS e outros eventos de febre amarela, e adaptado ao contexto de São Sebastião. As informações vacinais foram obtidas a partir da caderneta de vacinação e, quando ausente, consultadas no SI-PNI. 

O banco de dados secundários, gerado a partir da investigação de campo e encaminhado à SES-DF, foi anonimizado, agregado e utilizado para as análises apresentadas neste estudo.

### Viés

Potenciais fontes de viés incluíram: memória (lembrança imprecisa sobre vacinação pregressa); informação (ausência de registros vacinais, que pode ter subestimado a cobertura vacinal); resposta (a leitura das opções de resposta em algumas perguntas pode ter influenciado a frequência de acertos); e aferição (uso de diferentes entrevistadores pode ter introduzido variação na coleta).

Para reduzir esses riscos, foram adotadas medidas de mitigação: treinamento e padronização da equipe de campo, dupla checagem das informações coletadas, uso preferencial de registros oficiais, sorteio para seleção do respondente de CAP e acompanhamento diário da consistência dos dados.

### Tamanho do estudo

O tamanho da amostra foi calculado considerando amostragem probabilística por conglomerados do tipo 30x7, a partir de 120 setores censitários do IBGE incluindo áreas urbanas e rurais, intervalo de confiança de 95% (IC95%), efeito de desenho (ED) esperado de 2,0 e perda de 30,0%, resultando em 210 entrevistas previstas. A seleção foi realizada em três estágios: 1) sorteio dos conglomerados a partir dos setores censitários; 2) seleção sistemática dos domicílios; e 3) sorteio de um morador elegível por domicílio. Por fim, foram realizadas 210 entrevistas em 30 conglomerados, contemplando a distribuição planejada.

### Métodos estatísticos

As análises consideraram o plano amostral complexo. Para o inquérito vacinal, utilizou-se estatística descritiva, sendo calculadas frequências absolutas e relativas, medida de tendência central e intervalo de confiança de 95% (IC95%). Para o estudo CAP, utilizou-se estatística analítica, tendo a razão de prevalência (RP) como medida de associação, com IC95%. Foram apresentadas estimativas brutas. O erro padrão (EP) foi apresentado como medida de precisão.

O processamento dos dados foi realizado por meio dos programas Epi Info 7.2.4.0, Google Earth Pro 7.3.3.7786, Microsoft Office 2016, Sorteador e QGIS 3.4.13.

## Resultados

**Figura 1:**
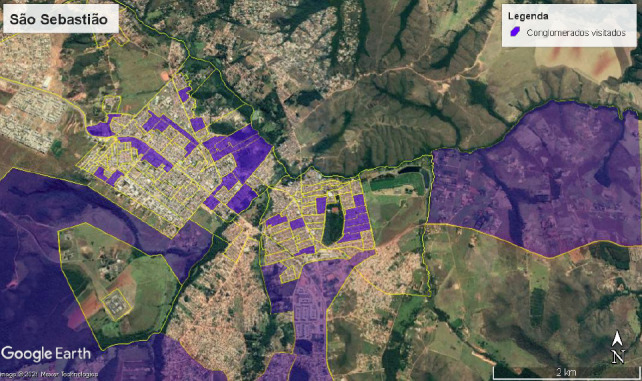
Distribuição geográfica dos conglomerados visitados em áreas urbanas e rurais. São Sebastião, Distrito Federal, 2020 (n=30)

Foram visitados 30 conglomerados em São Sebastião, Distrito Federal, entre novembro e dezembro de 2020, sendo utilizados quatro conglomerados reserva na ausência de domicílios ou de recente redefinição territorial para outra região administrativa. Do total de 210 entrevistas realizadas, 189 (90,0%) foram provenientes da zona urbana e 21 (10,0%) da zona rural (Figura 1). 

A amostra foi composta majoritariamente por pessoas do sexo feminino (63,8%; IC95% 57,30; 70,30) e que se autodeclararam de raça/cor da pele parda (60,0%; IC95% 53,40; 66,60). A escolaridade predominante foi o ensino médio completo (32,9%), seguida do ensino fundamental incompleto (25,2%). A média de idade dos participantes foi de 34,9 anos (±1,5) e o tempo médio de residência em São Sebastião foi de 12,9 anos (±0,9) (Tabela 1). 

**Tabela 1:** Intervalo de confiança de 95% (IC95%), efeito de desenho (ED), média e erro padrão (EP) das características sociodemográficas da população estudada. São Sebastião, Distrito Federal, 2020 (n=210)

Variáveis	n (%)	IC95%	ED
**Sexo**			
Feminino	134 (63,8)	57,30; 70,30	0,9
Masculino	76 (36,2)	29,70; 42,70	
**Raça/cor da pele autodeclarada**			
Parda	126 (60,0)	53,40; 66,60	0,8
Branca	39 (18,6)	12,90; 24,20	
Preta	35 (16,7)	11,60; 21,70	
Amarela	6 (2,9)	0,70; 5,00	
Indígena	1 (0,5)	-0,50; 1,50	
Ignorado	3 (1,4)	-0,20; 3,10	
**Escolaridade**			
Superior completo	25 (11,9)	7,90; 15,90	0,9
Superior incompleto	16 (7,6)	4,30; 10,90	
Médio completo	69 (32,9)	24,80; 40,90	
Médio incompleto	24 (11,4)	7,60; 15,20	
Fundamental completo	16 (7,6)	3,70; 11,50	
Fundamental incompleto	53 (25,2)	19,20; 31,30	
Analfabeto	3 (1,4)	-0,20; 3,10	
Ignorado	4 (1,9)	0,10; 3,70	
	**Média (EP)**	**IC95%**	
Idade (em anos)	34,9 (±1,5)	31,90; 38,10	
Tempo de residência (em anos) (n=205)	12,9 (±0,9)	11,10; 14,80	

Quanto à situação ocupacional, 47,1% (IC95% 40,3; 54,0) informaram não possuir trabalho remunerado. Em relação à renda familiar mensal, 52,4% relataram rendimentos entre um e dois salários mínimos, enquanto 89,5% informaram não ser beneficiários do programa Bolsa Família. 

A cobertura vacinal verificada por meio de comprovação documental foi de 54,3% (IC95% 47,70; 60,90), sendo a maioria dos registros provenientes da caderneta de vacinação (93,9%). Entre as pessoas vacinadas, 32,9% (IC95% 27,80; 37,90) apresentaram uma dose registrada. Observou-se que todas as crianças menores de cinco anos estavam vacinadas. 

A maioria dos participantes (85,7%) declarou utilizar exclusivamente os serviços públicos de saúde. Sobre exposição a áreas de risco, 22,9% referiram frequentar áreas de mata ou cachoeira. 

Em relação às motivações para vacinação, 29,5% referiram ter buscado a vacina por iniciativa própria, visando proteção contra a doença, e 23,8% foram influenciados por campanhas de vacinação. Entre os não vacinados, os principais motivos foram barreiras no acesso, como unidades de saúde lotadas e contraindicações médicas. Do total de entrevistados, 48,6% não apresentaram a caderneta de vacinação no momento da entrevista, sendo a principal justificativa o extravio do documento (23,8%). Entre aqueles que apresentaram algum registro de vacinação, a maioria (93,9%) tinha a comprovação na própria caderneta de vacinação. Apenas 5,2% relataram dificuldades no acesso à vacina, como ausência de imunobiológico ou longas filas nos serviços de saúde. 

A vacinação ocorreu predominantemente em serviços públicos de saúde de São Sebastião (70,5%), sendo as unidades básicas de saúde os locais mais frequentes (77,6%). 

Praticamente todos os participantes (99,5%) afirmaram já ter ouvido falar sobre a febre amarela. As principais fontes de informação foram a televisão (59,0%), a internet (17,1%) e os profissionais de saúde (15,7%).

Quanto aos conhecimentos sobre a doença, 61,9% dos entrevistados souberam identificar a febre amarela como uma enfermidade e 48,6% reconheceram corretamente o modo de transmissão. Apenas 30,9% indicaram a vacinação como forma de prevenção, e 43,3% não souberam informar o número de doses recomendadas. A maioria sabia que a vacina é preventiva (85,7%) e que o público-alvo inclui indivíduos de 9 meses a 59 anos de idade (63,8%).

As atitudes diante da vacinação foram majoritariamente adequadas: 97,1% aconselhavam seus familiares a se vacinar, embora apenas 16,7% tenham buscado informações sobre a vacina. Em relação às práticas, 54,3% apresentaram comprovação vacinal. De acordo com a classificação geral dos indicadores, 70,0% apresentaram conhecimentos inadequados, 87,1% atitudes adequadas e 54,3% práticas adequadas (Tabela 2).

**Tabela 2:** Classificação de conhecimentos, atitudes e práticas sobre vacinação contra febre amarela em adequado e inadequado com intervalo de confiança de 95% (IC95%) e efeito de desenho (ED). São Sebastião, Distrito Federal, 2020 (n=210)

	Conhecimentos			Atitudes			Práticas		
	n (%)	IC95%	ED	n (%)	IC95%	ED	n (%)	IC95%	ED
Adequado	63 (30,0)	22,90; 37,10	1,2	183 (87,1)	82,00; 92,30	1,2	114 (54,3)	47,70; 60,90	0,9
Inadequado	147 (70,0)	62,90; 77,10		27 (12,9)	7,70; 17,90		96 (45,7)	39,10; 52,30	

**Figura 2:**
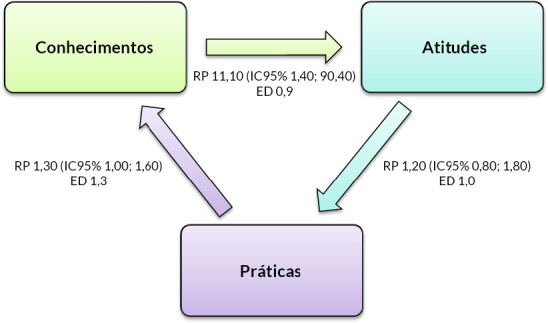
Razão de prevalência (RP), intervalo de confiança de 95% (IC95%) e efeito de desenho (ED) segundo conhecimentos, atitudes e práticas sobre vacinação contra febre amarela. São Sebastião, Distrito Federal, 2020 (n=210)

Ao estratificar os indicadores por variáveis sociodemográficas (sexo, escolaridade e renda familiar), não se observaram associações estatisticamente significativas (Tabela 3). Por outro lado, ao analisar as relações entre os próprios indicadores, observou-se que a prevalência de atitudes adequadas entre pessoas com conhecimentos adequados foi 11,10 (IC95% 1,40; 90,40) vezes maior do que entre aquelas com conhecimentos inadequados. Não se observou associação significativa entre atitudes e práticas, nem entre conhecimentos e práticas (Figura 2). 

**Tabela 3:** Razão de prevalência (RP), intervalo de confiança de 95% (IC95%) e efeito de desenho (ED) de conhecimentos, atitudes e práticas sobre vacinação contra febre amarela segundo sexo, escolaridade e renda familiar mensal. São Sebastião, Distrito Federal, 2020 (n=210)

	Conhecimentos				Atitudes				Práticas			
	Adequado	Inadequado	RP		Adequado	Inadequado	RP		Adequado	Inadequado	RP	
	n (%)	n (%)	(IC95%)	ED	n (%)	n (%)	(IC95%)	ED	n (%)	n (%)	(IC95%)	ED
**Sexo**												
Feminino (n=134)	38 (28,4)	96 (71,6)	0,90 (0,80; 1,20)	1,3	119 (88,8)	15 (11,2)	1,40 (0,60; 3,50)	0,9	77 (57,5)	57 (42,5)	1,20 (0,90; 1,70)	0,8
Masculino (n=76)	25 (32,9)	51 (67,1)			64 (84,2)	12 (15,8)			37 (48,7)	39 (51,3)		
**Escolaridade**												
Alta (n=110)	36 (32,7)	74 (67,3)	1,10 (0,90; 1,30)	1,2	100 (90,9)	10 (9,1)	1,80 (0,80; 4,20)	1,4	60 (54,5)	50 (45,5)	1,00 (0,70; 1,40)	0,8
Baixa (n=96)	25 (26,1)	71 (73,9)			80 (83,3)	16 (16,7)			52 (54,2)	44 (45,8)		
**Renda familiar mensal**												
Média/alta (n=56)	20 (35,7)	36 (64,3)	1,10 (0,90; 1,40)	0,8	49 (87,5)	7 (12,5)	1,00 (0,40; 2,30)	0,9	27 (48,2)	29 (51,8)	0,80 (0,50; 1,20)	1,6
Baixa (n=142)	40 (28,2)	102 (71,8)			124 (87,3)	18 (12,7)			82 (57,7)	60 (42,3)		

## Discussão

A cobertura vacinal contra febre amarela encontrada neste estudo (54,3%) foi abaixo da meta preconizada pelo Ministério da Saúde (95,0%), resultado preocupante diante de uma epizootia confirmada , considerada evento sentinela prioritário para vigilância da doença e risco potencial de reurbanização ^2^ ^,^ ^4^ ^,^ ^11^. Esse cenário reforça a relevância de investigações epidemiológicas oportunas, principalmente conduzidas por programas de treinamento em epidemiologia de campo como o Programa de Treinamento em Epidemiologia Aplicada aos Serviços do Sistema Único de Saúde (EpiSUS).

O perfil sociodemográfico dos participantes foi caracterizado por maioria feminina, adulta, de raça/cor da pele parda, com escolaridade até o ensino médio e baixa renda. Esse padrão, já descrito em outros estudos, reflete barreiras sociais e estruturais no acesso à imunização ^12^ ^,^ ^13^. A ausência de vínculo empregatício e a não participação em programas sociais podem indicar maior vulnerabilidade econômica, associada a menor acesso a serviços de saúde e, consequentemente, à vacinação. Tais achados demonstram a relevância das ações de saúde pública e vacinação em massa voltadas a essas populações.

Embora os serviços públicos tenham sido a principal fonte de atendimento, persistiram dificuldades operacionais que comprometem o acesso, como horários restritos, distância das unidades e possíveis falhas no registro vacinal. A baixa posse ou extravio da caderneta, observada em quase metade dos participantes, pode ter limitado a atualização e comprovação vacinal, algo também relatado em outras populações ^14^ ^,^ ^15^.

As campanhas de vacinação foram apontadas como principal motivo para imunização, confirmando sua relevância como estratégia de prevenção. Entretanto, a descontinuidade dessas campanhas pode reduzir a percepção de risco, especialmente em áreas urbanas com baixa divulgação de informações, favorecendo a circulação viral ^16^ ^,^ ^17^.

No componente de conhecimentos, ainda que a maioria dos entrevistados tivesse ouvido falar da febre amarela, apenas 30,9% identificaram corretamente a vacina como principal forma de prevenção, e muitos desconheciam o número de doses recomendadas. Lacunas semelhantes foram relatadas em estudos na África e na América Latina, mesmo entre vacinados ^18^ ^-^ ^20^. As atitudes, por outro lado, mostraram-se mais favoráveis: a maioria considerou a doença grave, reconheceu a eficácia da vacina e recomendou a imunização a familiares. Contudo, práticas inadequadas persistiram, refletindo a distância entre intenção, comportamento e ação, realidade também descrita em outros estudos de conhecimentos, atitudes e práticas ^21^ ^,^ ^22^.

Foi identificada associação positiva entre conhecimentos adequados e atitudes pró-vacinação, em concordância com a literatura sobre a influência do conhecimento no comportamento preventivo ^20^. Por outro lado, variáveis como sexo, renda e escolaridade não apresentaram associação estatisticamente significativa, diferindo de achados de outros contextos. A homogeneidade socioeconômica da população estudada e o tamanho amostral podem explicar essa divergência ^23^ ^-^ ^25^.

O estudo apresenta algumas limitações. O viés de memória pode ter afetado a acurácia das respostas, especialmente quanto à vacinação pregressa. A ausência de registros pode ter subestimado a cobertura real. A leitura de opções de resposta em algumas perguntas pode ter influenciado a frequência de acertos, e o uso de diferentes entrevistadores, ainda que treinados, pode ter introduzido viés de aferição.

Apesar dessas limitações, os achados contribuíram para a intensificação da vacinação em São Sebastião, após a investigação. Este estudo destaca a utilidade de epizootias em primatas não humanos como eventos sentinela para mobilização antecipada das ações de vigilância em saúde, como o monitoramento precoce da circulação viral e implementação de respostas intersetoriais ^1^ ^,^ ^3^ ^,^ ^26^. Durante o período investigado, não foram registrados casos humanos de febre amarela.

Recomendou-se à Secretaria de Saúde do Distrito Federal intensificar a vacinação contra a febre amarela em São Sebastião, com reforço da busca ativa e ampliação do acesso da população aos pontos de vacinação; desenvolver campanhas educativas sobre febre amarela em meios de maior alcance (televisão e internet); fortalecer as ações de vacinação junto às Equipes de Saúde da Família; inserir o tema febre amarela no Programa Saúde na Escola; capacitar profissionais de saúde quanto às atualizações do esquema vacinal; estimular a população a manter a caderneta de vacinação atualizada; e monitorar periodicamente a cobertura vacinal, mantendo os registros atualizados nos sistemas de informação.

Conclui-se que a cobertura vacinal foi inferior à meta estabelecida e que persistem lacunas relevantes nos conhecimentos sobre a febre amarela e sua prevenção, embora atitudes favoráveis predominem. Esses resultados podem subsidiar a formulação de estratégias educativas e de comunicação, integradas à atenção primária, para ampliar o acesso e a adesão à vacinação. O estudo reforça o papel do EpiSUS e de investigações de campo como ferramentas estratégicas para respostas rápidas e baseadas em evidências diante de eventos sentinela e potenciais emergências em saúde pública.

## Data Availability

Os dados da pesquisa podem ser obtidos mediante solicitação à Secretaria de Saúde do Distrito Federal por meio do link: https://www.participa.df.gov.br/pages/registro-manifestacao/relato.
