# Supplementary material for: Cobertura vacinal e conhecimentos, atitudes e práticas sobre vacinação contra febre amarela: estudo transversal durante epizootia em São Sebastião, Distrito Federal, 2020
Source: Epidemiol Serv Saude. 2025 Nov 21;35:e20250739. [Article in Portuguese] doi: 10.1590/S2237-96222026v35e20250739.pt (PMC12641599; doi:10.1590/S2237-96222026v35e20250739.pt)
Supplement: Translation

# Translation: Yellow fever vaccination coverage and knowledge, attitudes and practices: a cross-sectional study during an epizootic event in São Sebastião, Federal District, 2020

**DOI:** 10.1590/S2237-96222026v35e20250739.en

**Authors:** Leonardo José Alves de Freitas, Lairton Souza Borja, Danielle Gonçalves Figueiredo, Fabiano dos Anjos Pereira Martins, Priscilleyne Ouverney Reis, Aline Ruben Cardoso Fernandes Caixeta, Ana Júlia Silva e Alves

**Affiliations:** 1 Ministério da Saúde, Secretaria de Vigilância em Saúde e Ambiente, Programa EpiSUS, Brasília, DF, Brazil; 2 Secretaria de Saúde do Distrito Federal, Núcleo de Vigilância Epidemiológica Leste, Brasília, DF, Brazil; 3 Secretaria de Saúde do Distrito Federal, Subsecretaria de Vigilância à Saúde, Brasília, DF, Brazil; 4 Secretaria de Saúde do Distrito Federal, Diretoria de Vigilância Epidemiológica, Brasília, DF, Brazil; 5 Secretaria de Saúde do Distrito Federal, Diretoria de Vigilância Ambiental, Brasília, DF, Brazil

**Keywords:** Yellow Fever, Public Health Surveillance, Vaccines, Epidemiology, Cross-Sectional Studies

## Abstract

**Objective:**

To estimate the yellow fever vaccination coverage and describe the knowledge, attitudes and practices (KAP) of the population residing in São Sebastião, Federal District, Brazil, after confirmation of a non-human primate epizootic event in the region.

**Methods:**

Cross-sectional study consisting of two surveys, a vaccination coverage survey and a KAP survey, between November and December 2020. Probabilistic cluster sampling (30x7) was adopted. Individuals with the yellow fever vaccine recorded in an immunization record or in the National Immunization Program Information System were considered vaccinated. Complex analysis and descriptive statistics, with absolute and relative frequencies, measures of central tendency and accuracy were used. The KAP study employed analytical statistics, adopting prevalence ratio as measure of association and 95% confidence intervals (95%CI).

**Results:**

In total, 210 interviews were conducted. Vaccination coverage was 54.3% (95%CI 47.70; 60.90), with 93.9% of the records obtained from the immunization record. Of the participants, 63.8% were female and 60.0% self-declared as brown. Regarding the KAP component, 70.0% presented inadequate knowledge, 87.1% presented adequate attitudes and 54.3% presented adequate practices. The prevalence of adequate attitudes among people with adequate knowledge was 11.10 times higher than among those with inadequate knowledge.

**Conclusion:**

The study provided a description of the yellow fever vaccination coverage and KAP. The coverage found was below the 95.0% target established by the National Immunization Program, providing inputs that can support health surveillance and vaccination promotion initiatives.

**Ethical aspects**

This research respected the ethical principles, having obtained the following approval data:

Research Ethics Committee: National Research Ethics Committee

Opinion number: 5.013.116

Approval date: 3/10/2021

Certificate of Submission for Ethical Appraisal: 49195021.8.0000.0008

Informed Consent Form: In the field stage, participants signed the Informed Consent Form (ICF). For the analysis of secondary and anonymized data, National Research Ethics Committee granted the waiver of a new ICF.

## Introduction

Yellow fever is an acute arboviral disease, with worldwide importance, caused by a virus of the genus *Flavivirus*. Transmission to humans occurs mainly in the sylvatic cycle, through the bite of mosquitoes of the genera *Haemagogus* and *Sabethes*. Non-human primates (NHP) are considered the main hosts and amplifiers of the virus in the sylvatic cycle of the disease ^1^. Although Brazil has not recorded urban transmission since 1942, the occurrence of epizootic events near urbanized areas represents a risk of reurbanization of the disease and keeps public health agencies on alert ^2^.

Between July 2019 and June 2020, Brazil reported 976 suspected cases of yellow fever, with 19 confirmed cases and four deaths ^3^. In the same period, in the Federal District, there were 11 suspected cases in humans, all discarded. In November 2020, an epizootic event was confirmed in a NHP in the administrative region of São Sebastião ^4^, located in the eastern portion of the Brazilian capital. The episode coincided with the beginning of the seasonal period of the disease, from December to May, amid the COVID-19 pandemic ^1^.

Vaccination is the most effective yellow fever prevention measure. Despite that, vaccination coverage remains low in several regions of Brazil, especially in transition regions between urban and rural areas ^1^ ^,^ ^5^. Factors such as ignorance about the disease, negative attitudes towards the vaccine and insufficient prevention practices directly influence the population’s adherence to immunization against yellow fever, promoting vaccine hesitancy ^6^.

In addition to information-related barriers, health care services face operational challenges, such as occasional vaccine shortages, limited active search, and logistical difficulties in updating vaccine registries and records ^7^. Such deficiencies can compromise the effectiveness of immunization initiatives and favor the persistence of pockets susceptible to the yellow fever virus even in areas covered by vaccination campaigns. 

Despite the relevance of yellow fever in the Federal District, there is a lack of local studies describing the vaccination coverage and the knowledge, attitudes and practices (KAP) of the population, especially in vulnerable territories such as São Sebastião. This information gap reinforces the importance of epidemiological surveys to support and direct health surveillance initiatives and immunization promotion, for the purpose of prevention and intervention against infectious diseases.

In this context, the present study aimed to estimate the yellow fever vaccination coverage and describe the KAP of the population residing in São Sebastião, Federal District, Brazil, after confirmation of an NHP epizootic event in the region.

## Methods

### Study design

This was a population-based cross-sectional study, consisting of two surveys: a yellow fever vaccination coverage survey and a knowledge, attitudes and practices (KAP) survey in relation to the disease and vaccination. The study originated from a field epidemiological investigation carried out from November 25 to December 18, 2020.

### Context

On November 9, 2020, the Federal District Health Department (SES-DF) laboratory confirmed the death of a non-human primate due to yellow fever in the São José neighborhood, an urban area in the administrative region of São Sebastião. On November 18, 2020, SES-DF requested the support of the Department of Health Surveillance and Environment of the Ministry of Health (SVSA), through the Training Program in Epidemiology Applied to Services of the Unified Health System (EpiSUS)/Brazil Field Epidemiology Training Program (Brazil FETP), Advanced level, for field epidemiological investigation.

The investigation was carried out in the territory of São Sebastião, an administrative region of the Federal District that is located in the Eastern Health Region of Brasília, 23 km from Plano Piloto. The region has urban and rural characteristics, with an estimated population of 115,000 inhabitants in 2020, distributed in 120 census sectors. The territory had 14 basic health units (BHU) and 78.1% coverage by Family Health Teams. The fixed vaccination rooms were in BHU 1 and in the Penitentiary System, with other 12 BHUs carrying out itinerant vaccination on specific days. The surveys were conducted at the beginning of the yellow fever seasonal period, amid the COVID-19 pandemic.

### Participants

Sampling followed the probabilistic cluster method (30x7), recommended by the World Health Organization (8,9). In each randomly selected household, residents over nine months of age present at the time of the interview were invited to participate in the vaccination coverage survey. For the KAP survey, a resident aged 18 years or older was selected, defined by draw among the eligible people present in the household. Residents absent at the time of the visit and those who refused participation were excluded. 

The inclusion criteria required minimum residence time of three months in the region. Closed properties, commercial properties, churches, prison units and individuals unable to participate were excluded. The definitions of vaccinated, unvaccinated, complete and incomplete schedule, loss and refusal followed standardized criteria ^1^ ^,^ ^10^.

### Variables

Variables related to sociodemographic, vaccination and epidemiological characteristics were used. Sociodemographic variables included sex (female, male), self-declared race/skin color (brown, white, black, yellow, indigenous, ignored), education (complete higher education, incomplete higher education, complete secondary education, incomplete secondary education, complete elementary education, incomplete elementary education, illiterate, ignored), age (in years), residence time (in years), occupation, monthly household income and receipt of social benefits. 

The main variable of the study was vaccination coverage, defined according to the record of at least one dose of the yellow fever vaccine in the immunization record or in the National Immunization Program Information System (SI-PNI), according to the vaccination schedule of the Ministry of Health. Coverage was estimated as the ratio of respondents with proven vaccine record in relation to the total sample. Epidemiological variables were also analyzed, including access to health care services, visits to forest or waterfall areas, motivations and barriers to vaccination, place of vaccination and sources of information.

In the KAP survey, knowledge was assessed based on six questions related to the disease and the vaccine (what is yellow fever, how does transmission occur, forms of protection, purpose of the vaccine, target public and number of doses recommended for adults). Respondents who answered at least four of these questions correctly (66.7%) were classified as having adequate knowledge. Attitudes were measured by six questions on the disease’s perceived risk, prevention, search for information, intention to get vaccinated, trust in the vaccine safety, and incentive to vaccinate family members, being considered adequate when there were at least four positive answers. Practices were defined by vaccination status, being classified as adequate when the participant presented at least one dose recorded in an immunization record or in the SI-PNI.

Derived variables were used according to the classification of the Brazilian Institute of Geography and Statistics (IBGE). Education was categorized as low (illiterate, incomplete or complete elementary education, incomplete secondary education) and high (complete secondary education, incomplete or complete higher education). Monthly household income was categorized as low (up to two minimum monthly wages) and medium/high (from three to ten minimum monthly wages).

### Data sources and measurement

During the field investigation, face-to-face home interviews were conducted, applied with a previously tested and standardized semi-structured questionnaire. The instrument used was prepared by the research team, based on KAP instruments previously applied in surveys conducted within the scope of EpiSUS and other yellow fever events, and adapted to the context of São Sebastião. Vaccination information was obtained from the immunization record and, when lacking, consulted in the SI-PNI. 

The secondary database, generated from the field investigation and sent to SES-DF, was anonymized, aggregated and used for the analyses presented in this study.

### Bias

Potential sources of bias included: memory (inaccurate recall about previous vaccination); information (lack of vaccination records, which may have underestimated vaccination coverage); answer (the reading of the answer options in some questions may have influenced the frequency of correct answers); and measurement (use of different interviewers may have introduced variation in the collection).

To reduce these risks, we adopted mitigation measures: field team training and standardization, double checking of information collected, preferential use of official records, random selection of the KAP respondent, and daily monitoring of data consistency.

### Study size

Sample size was calculated considering 30x7 probabilistic cluster sampling, from 120 IBGE census sectors including urban and rural areas, 95% confidence interval (95%CI), expected design effect (Deff) of 2.0 and loss of 30.0%, resulting in 210 expected interviews. Selection was carried out in three stages: 1) drawing of clusters from the census sectors; 2) systematic selection of households; and 3) drawing of one eligible resident per household. Finally, 210 interviews were conducted in 30 clusters, meeting the planned distribution.

### Statistical methods

The analyses considered the complex sampling plan. For the vaccination survey, descriptive statistics were used, calculating absolute and relative frequencies, measures of central tendency and 95% confidence intervals (95%CI). For the KAP study, we used analytical statistics, with prevalence ratio (PR) as measure of association, with 95%CI. Raw estimates were presented. Standard error (SE) was presented as measure of accuracy.

Data processing used Epi Info 7.2.4.0, Google Earth Pro 7.3.3.7786, Microsoft Office 2016, Sorteador and QGIS 3.4.13.

## Results

Thirty clusters were visited in São Sebastião, Federal District, Brazil, between November and December 2020, and four reserve clusters were used in the absence of households or recent territorial redefinition for other administrative region. Of the total 210 interviews conducted, 189 (90.0%) respondents came from the urban area and 21 (10.0%) from the rural area (Figure 1). 

**Figure 1:** Geographic distribution of clusters visited in urban and rural areas. São Sebastião, Federal District, 2020 (n=30)

The sample consisted mostly of females (63.8%; 95%CI 57.30; 70.30) who self-declared as brown race/skin color (60.0%; 95%CI 53.40; 66.60). The predominant education level was complete secondary education (32.9%), followed by incomplete elementary education (25.2%). The participants’ mean age was 34.9 years (±1.5) and the mean residence time in São Sebastião was 12.9 years (±0.9) (Table 1). 

**Table 1:** 95% confidence interval (95%CI), design effect (Deff), mean and standard error (SE) of the sociodemographic characteristics of the studied population. São Sebastião, Federal District, 2020 (n=210)

Variables	n (%)	95%CI	Deff
**Sex**			
Female	134 (63.8)	57.30; 70.30	0.9
Male	76 (36.2)	29.70; 42.70	
**Self-declared race/skin color**			
Brown	126 (60.0)	53.40; 66.60	0.8
White	39 (18.6)	12.90; 24.20	
Black	35 (16.7)	11.60; 21.70	
Yellow	6 (2.9)	0.70; 5.00	
Indigenous	1 (0.5)	-0.50; 1.50	
Ignored	3 (1.4)	-0.20; 3.10	
**Education**			
Complete higher education	25 (11.9)	7.90; 15.90	0.9
Incomplete higher education	16 (7.6)	4.30; 10.90	
Complete secondary education	69 (32.9)	24.80; 40.90	
Incomplete secondary education	24 (11.4)	7.60; 15.20	
Complete elementary education	16 (7.6)	3.70; 11.50	
Incomplete elementary education	53 (25.2)	19.20; 31.30	
Illiterate	3 (1.4)	-0.20; 3.10	
Ignored	4 (1.9)	0.10; 3.70	
	Mean (SE)	95%CI	
Age (in years)	34.9 (±1.5)	31.90; 38.10	
Residence time (in years) (n=205)	12.9 (±0.9)	11.10; 14.80	

As for the occupational status, 47.1% (95%CI 40.3; 54.0) reported not having paid work. Regarding monthly household income, 52.4% reported income between one and two minimum monthly wages, while 89.5% reported not being beneficiaries of the Bolsa Família program. 

Vaccination coverage verified through documentary evidence was 54.3% (95%CI 47.70; 60.90), with most records obtained from the immunization record (93.9%). Among vaccinated people, 32.9% (95%CI 27.80; 37.90) had a recorded dose. It was observed that all children under the age of five were vaccinated. 

Most participants (85.7%) reported using public health care services exclusively. Regarding exposure to risk areas, 22.9% reported attending areas of forest or waterfall. 

Regarding the motivations for vaccination, 29.5% reported having sought the vaccine on their own initiative, aiming at protection against the disease, and 23.8% were influenced by vaccination campaigns. Among unvaccinated people, the main reasons were barriers to access, such as crowded health care facilities, and medical contraindications. Of the total number of respondents, 48.6% did not present the immunization record at the time of the interview, the main justification being the loss of the document (23.8%). Among those who presented some vaccination record, the majority (93.9%) had the proof in the immunization record itself. Only 5.2% reported difficulties in accessing the vaccine, such as lack of immunobiological agents or long queues in health care services. 

Vaccination occurred predominantly in public health care services in São Sebastião (70.5%), with basic health units being the most frequent locations (77.6%). 

Virtually all participants (99.5%) said they had heard about yellow fever. The main sources of information were television (59.0%), the internet (17.1%) and health care professionals (15.7%).

Regarding knowledge about the disease, 61.9% of respondents knew how to identify yellow fever as a disease and 48.6% correctly recognized the mode of transmission. Only 30.9% indicated vaccination as a form of prevention, and 43.3% were unable to inform the number of recommended doses. Most knew that the vaccine is preventive (85.7%) and that the target public includes individuals aged from 9 months to 59 years (63.8%).

Attitudes towards vaccination were mostly adequate: 97.1% advised their family members to get vaccinated, although only 16.7% had sought information about the vaccine. Regarding the practices, 54.3% presented proof of vaccination. According to the general classification of the indicators, 70.0% presented inadequate knowledge, 87.1% adequate attitudes and 54.3% adequate practices (Table 2).

**Table 2:** Classification of knowledge, attitudes and practices on yellow fever vaccination into adequate and inadequate with 95% confidence interval (95%CI) and design effect (Deff). São Sebastião**, Federal District,** 2020 (n=210)

	Knowledge			Attitudes			Practices		
	n (%)	95%CI	Deff	n (%)	95%CI	Deff	n (%)	95%CI	Deff
Adequate	63 (30.0)	22.90; 37.10	1.2	183 (87.1)	82.00; 92.30	1.2	114 (54.3)	47.70; 60.90	0.9
Inadequate	147 (70.0)	62.90; 77.10		27 (12.9)	7.70; 17.90		96 (45.7)	39.10; 52.30	

When stratifying the indicators by sociodemographic variables (sex, education and household income), no statistically significant associations were observed (Table 3). On the other hand, when analyzing the relations between the indicators, it was observed that the prevalence of adequate attitudes among people with adequate knowledge was 11.10 (95%CI 1.40; 90.40) times higher than among those with inadequate knowledge. There was no significant association between attitudes and practices, nor between knowledge and practices (Figure 2).

**Table 3:** Prevalence ratio (PR), 95% confidence interval (95%CI) and design effect (Deff) of knowledge, attitudes and practices on yellow fever vaccination according to sex, education and monthly household income. São Sebastião**, Federal District,** 2020 (n=210)

	Knowledge				Attitudes				Practices			
	Adequate	Inadequate	PR		Adequate	Inadequate	PR		Adequate	Inadequate	PR	
	n (%)	n (%)	(95%CI)	Deff	n (%)	n (%)	(95%CI)	Deff	n (%)	n (%)	(95%CI)	Deff
**Sex**												
Female (n=134)	38 (28.4)	96 (71.6)	0.90 (0.80; 1.20)	1.3	119 (88.8)	15 (11.2)	1.40 (0.60; 3.50)	0.9	77 (57.5)	57 (42.5)	1.20 (0.90; 1.70)	0.8
Male (n=76)	25 (32.9)	51 (67.1)			64 (84.2)	12 (15.8)			37 (48.7)	39 (51.3)		
**Education**												
High (n=110)	36 (32.7)	74 (67.3)	1.10 (0.90; 1.30)	1.2	100 (90.9)	10 (9.1)	1.80 (0.80; 4.20)	1.4	60 (54.5)	50 (45.5)	1.00 (0.70; 1.40)	0.8
Low (n=96)	25 (26.1)	71 (73.9)			80 (83.3)	16 (16.7)			52 (54.2)	44 (45.8)		
**Monthly household income**												
Medium/high (n=56)	20 (35.7)	36 (64.3)	1.10 (0.90; 1.40)	0.8	49 (87.5)	7 (12.5)	1.00 (0.40; 2.30)	0.9	27 (48.2)	29 (51.8)	0.80 (0.50; 1.20)	1.6
Low (n=142)	40 (28.2)	102 (71.8)			124 (87.3)	18 (12.7)			82 (57.7)	60 (42.3)		

**Figure 2:** Prevalence ratio (PR), 95% confidence interval (95%CI) and design effect (Deff) according to knowledge, attitudes and practices on yellow fever vaccination. São Sebastião, Federal District, 2020 (n=210)

## Discussion

The yellow fever vaccination coverage found in this study (54.3%) was below the Ministry of Health target (95.0%), a worrying result in the face of a confirmed epizootic event, considered a priority sentinel event for disease surveillance and potential risk of reurbanization ^2^ ^,^ ^4^ ^,^ ^11^. This situation reinforces the relevance of timely epidemiological investigations, especially conducted by field epidemiology training programs such as the Training Program in Epidemiology Applied to Services of the Unified Health System (EpiSUS).

The participants’ sociodemographic profile was characterized by majority of female, adult, brown race/skin color, with education up to secondary education and low income. This pattern, already described in other studies, reflects social and structural barriers to access to immunization ^12^ ^,^ ^13^. Lack of employment and non-participation in social programs may indicate higher economic vulnerability, associated with lower access to health care services and, consequently, to vaccination. These findings demonstrate the relevance of public health and mass vaccination initiatives geared toward these populations.

Although public services were the main source of care, there are persisting operational difficulties that compromise access, such as restricted hours, distance from the units and possible failures in the vaccination record. The low possession or loss of the immunization record booklet, observed in almost half of the participants, may have limited the update and proof of vaccination, which has also been reported in other populations ^14^ ^,^ ^15^.

Vaccination campaigns were indicated as the main reason for immunization, confirming their relevance as a prevention strategy. However, the discontinuity of these campaigns may reduce the perceived risk, especially in urban areas with low dissemination of information, favoring viral circulation ^16^ ^,^ ^17^.

In the knowledge component, although most respondents had heard of yellow fever, only 30.9% correctly identified the vaccine as the main form of prevention, and many were unaware of the number of recommended doses. Similar gaps have been reported in studies in Africa and Latin America, even among vaccinated people ^18^ ^-^ ^20^. Attitudes, on the other hand, were more favorable: most considered the disease serious, recognized the effectiveness of the vaccine and recommended immunization to family members. However, inadequate practices persisted, reflecting the distance between intention, behavior and action, a situation also described in other studies of knowledge, attitudes and practices ^21^ ^,^ ^22^.

A positive association was found between adequate knowledge and pro-vaccination attitudes, consistently with the literature on the influence of knowledge on preventive behavior ^20^. On the other hand, variables such as sex, income and education did not present a statistically significant association, differing from findings from other contexts. The socioeconomic homogeneity of the studied population and the sample size may explain this divergence ^23^ ^-^ ^25^.

The study presents some limitations. Memory bias may have affected the accuracy of answers, especially for previous vaccination. The lack of records may have underestimated the actual coverage. The reading of answer options in some questions may have influenced the frequency of correct answers, and the use of different interviewers, even if trained, may have introduced measurement bias.

Despite these limitations, the findings contributed to the intensification of vaccination in São Sebastião, after the investigation. The study emphasizes the usefulness of non-human primate epizootics as sentinel events for early implementation of health surveillance initiatives, such as early monitoring of viral circulation and mobilization of intersectoral responses ^1^ ^,^ ^3^ ^,^ ^26^. During the investigated period, no human cases of yellow fever were recorded.

The Federal District Department of Health was recommended to intensify yellow fever vaccination in São Sebastião, reinforcing active search and expanding the population’s access to vaccination points; develop educational campaigns on yellow fever in mass media (television and internet); strengthen vaccination initiatives with Family Health Teams; include the yellow fever topic in the School Health Program; train health care professionals on updates of the vaccination schedule; encourage the population to keep the immunization record booklet updated; and periodically monitor vaccination coverage, keeping records updated in information systems.

It is concluded that vaccination coverage was below the established target and that relevant gaps remain in knowledge about yellow fever and its prevention, although favorable attitudes predominate. These results can support the formulation of educational and communicational strategies, integrated with primary health care, to increase access and adherence to vaccination. The study reinforces the role of EpiSUS and field investigations as strategic tools for rapid, evidence-based responses to sentinel events and potential public health emergencies.

## Data Availability

The research data can be obtained upon request to the Health Department of the Federal District, Brazil through the link: https://www.participa.df.gov.br/pages/registro-manifestacao/relato.
